# Incisional Negative-Pressure Wound Therapy Versus Standard Dressing for the Prevention of Surgical Site Complications Following Radical Cystectomy

**DOI:** 10.3390/jpm15120581

**Published:** 2025-11-30

**Authors:** Fabio Traunero, Arianna Biasatti, Giulio Rossin, Andrea Piasentin, Federico Zorzi, Michele Rizzo, Paolo Umari, Tommaso Cai, Alessandro Zucchi, Giovanni Liguori

**Affiliations:** 1 Urological Clinic, Department of Medical, Surgical and Health Sciences, Cattinara Hospital, University of Trieste, 34149 Trieste, Italy; 2 Department of Urology, Ospedale Santa Maria Della Misericordia di Udine, 33100 Udine, Italy; 3 Department of Urology, Santa Chiara Hospital, 38121 Trento, Italy; 4 Department of Urology, University of Pisa, 56122 Pisa, Italy

**Keywords:** surgical site complications, PICO^®^ system, iNPWT, radical cystectomy, open surgery

## Abstract

**Background/Objectives:** Radical cystectomy (RC) is a complex urologic procedure that, when performed using an open surgical approach, carries a high risk of surgical site complications (SSCs), which can lead to prolonged recovery, increased healthcare costs, and higher morbidity. Incisional negative-pressure wound therapy (iNPWT) has demonstrated benefits in enhancing wound healing in various surgical settings, but its effectiveness in the context of RC remains underexplored. This study aimed to evaluate the impact of iNPWT on the incidence of SSCs following RC compared to standard surgical dressings using the PICO^®^ system. **Methods:** A cohort of 146 patients who underwent RC between 2015 and 2021 was divided into two groups: those treated with standard dressings (Group 1, *n* = 80) and those who received iNPWT (Group 2, *n* = 66). Patient-related, surgical, and pathological variables were compared between the groups. Categorical variables were analyzed using the chi-square or Fisher’s exact test, while continuous variables were assessed using Student’s *t*-test. Univariate logistic regression models were applied to evaluate the association between iNPWT use and 90-day SSCs, as well as to identify risk factors for complications. **Results:** Group 2 (iNPWT) had a higher prevalence of chronic comorbidities, including chronic kidney disease, but fewer active smokers compared to Group 1. Higher body mass index, prolonged operative time, and uncontrolled diabetes were significantly associated with the development of SSCs. The incidence of SSCs within 90 days was significantly lower in the iNPWT group (7.6%) compared to the standard dressing group (22.5%) (*p* = 0.03). iNPWT use was associated with a substantially reduced risk of SSCs (OR: 0.282), demonstrating a protective effect. **Conclusions:** Prophylactic application of iNPWT following RC significantly reduced the incidence of surgical site complications compared to standard dressing. These findings support the potential of iNPWT as a valuable adjunct in perioperative wound management. While encouraging, these results warrant validation in prospective, randomized studies. Tailored postoperative strategies and identification of patient-specific risk factors remain essential components in SSC prevention and reflect the growing importance of precision medicine in surgical oncology.

## 1. Introduction

Bladder cancer ranks among the most prevalent malignancies and is the tenth most common cancer globally [1]. Radical cystectomy (RC) is the gold-standard treatment for muscle-invasive bladder cancer and selected high-risk non-muscle-invasive cases and may be performed via open, laparoscopic, or robot-assisted approaches [2]. Despite advances in surgical technique and perioperative pathways, RC remains associated with substantial morbidity. Surgical site complications (SSCs) after RC—such as hematoma, seroma, wound dehiscence, and superficial or deep infection—are frequent and clinically consequential, leading to prolonged hospitalization, delayed recovery, and greater costs [3,4,5]. Reported SSC rates vary across series, but estimates range from approximately 18% to 33% [4].

Incisional negative-pressure wound therapy (iNPWT) is a closed-incision intervention that applies subatmospheric pressure to optimize perfusion, reduce edema, manage exudate, and stabilize the incision microenvironment. Evidence for iNPWT has grown in colorectal and other abdominal procedures, yet dedicated data for RC are limited, and few studies have focused on single-use systems such as the PICO^®^ device [5,6,7,8,9]. Given the distinctive features of RC—urinary diversion, long operative times, and frequent multimorbidity—this population may represent a subgroup that could particularly benefit from selective application of iNPWT.

Beyond aggregate efficacy, a central question is that of how to deploy iNPWT in a way that contributes to personalized or precision medicine. Our working hypothesis was that risk stratification—based on readily measurable factors such as body mass index (BMI), diabetes control, chronic kidney disease (CKD), and operative duration—could identify patients most likely to benefit from prophylactic iNPWT. We therefore compared outcomes between patients receiving conventional dressings and those managed with iNPWT after open RC. We interpret the findings through an exploratory, hypothesis-generating, precision-medicine lens, emphasizing targeted postoperative wound management while acknowledging that allocation to iNPWT was time-based rather than risk-based and thus does not constitute a prospective test of a risk-stratified strategy.

## 2. Materials and Methods

We performed a retrospective cohort study of consecutive patients who underwent open RC at a single academic center. From January 2015 to April 2019, patients routinely received conventional postoperative dressings (Group 1). From May 2019 to June 2021, prophylactic iNPWT with the PICO^®^ system (Smith & Nephew, London, UK) was introduced and used in consecutive cases (Group 2). All operations were conducted by a single high-volume surgeon. In all cases, surgical access was obtained via a lower midline laparotomy. All suitable RC cases were included, regardless of the urinary diversion technique employed. Patients with incomplete follow-up or those who required unplanned reoperation for non-wound-related causes within one month were excluded. The study was approved by the Institutional Review Board. All included patients provided informed consent for the use of their clinical data for research purposes. Perioperative protocols were standardized. Antimicrobial prophylaxis was administered within one hour of incision and discontinued within 24 h postoperatively. Skin preparation used a chlorhexidine–alcohol scrub. Gloves were changed after completion of the anastomosis. Fascial closure was performed with continuous 1–0 polydioxanone sutures; the subcutaneous layer was approximated with interrupted 3–0 polyglactin (Vicryl) sutures; the skin was closed with clips. To ensure comparability in wound healing, no suction drain was placed in the subcutaneous tissue regardless of BMI. In the iNPWT group, the PICO^®^ dressing was applied over the intact incision in the operating room and maintained for one week. Care was taken to separate the device from any urinary stoma to avoid contamination. Controls received standard adherent gauze dressings.

**Figure 1:**
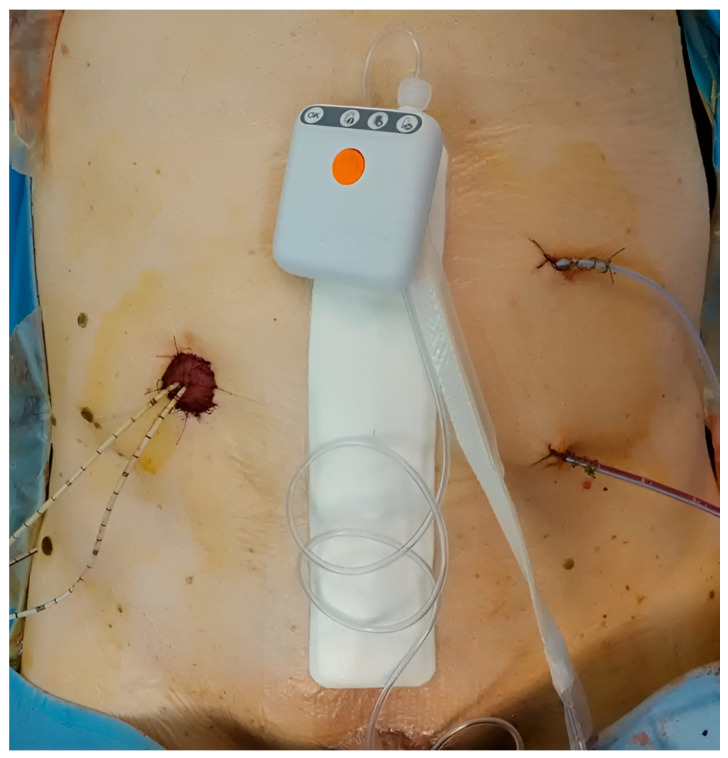
iNPWT dressing (PICO^®^ system) after open radical cystectomy.

The exposure of interest was prophylactic application of iNPWT (PICO^®^) to the closed midline laparotomy incision (Figure 1). The comparator was standard dressing. The decision to employ iNPWT followed service adoption and was not based on predefined selection criteria; patients were enrolled consecutively within each era.

The primary endpoint was the incidence of SSCs requiring intervention within 90 days of surgery, categorized according to criteria as hematoma, seroma, partial or complete dehiscence (with or without infection), and superficial or deep incisional infection [10]. Secondary outcomes included hospital length of stay and identification of patient- or procedure-related risk factors for SSCs.

Demographics and lifestyle factors included age, sex, BMI, smoking status (active if current or ceased within the prior year), and alcohol use. Comorbidities were extracted from medical records and summarized using the Charlson Comorbidity Index (CCI); anesthetic risk was classified by American Society of Anesthesiologists (ASA) category. We recorded the presence of CKD and diabetes mellitus (with attention to preoperative control), prior abdominal surgery, and relevant medications. Surgical variables comprised operative time, estimated blood loss, transfusion requirements, and urinary diversion type (continent vs. incontinent). Pathology variables included histology, grade, and pathological tumor stage according to the pTNM system [11].

Patients were assessed clinically at approximately 30 days and again around 90 days postoperatively. At each visit, the incision was examined to document SSCs, aesthetic appearance, and any late-presenting issues.

Categorical variables were compared using the chi-square or Fisher’s exact test as appropriate. Continuous variables were compared using Student’s *t*-test. We fitted multivariable logistic regression models to estimate odds ratios (ORs) and 95% confidence intervals for 90-day SSCs. Covariates were pre-specified a priori based on clinical relevance and observed baseline imbalances: treatment group (iNPWT vs. standard), BMI (continuous) and obesity (BMI ≥ 30), smoking status (active vs. former/never), CKD, diabetes, prior laparotomic abdominal surgery, operative time (continuous and ≥300 min), and a temporal indicator for the adoption era (pre–May 2019 vs. May 2019–June 2021) to mitigate era effects. Model assumptions and multicollinearity were evaluated using standard diagnostics; two-sided *p* ≤ 0.05 defined statistical significance. Analyses were conducted in IBM SPSS Statistics version 24.0.0.1 (IBM Corp., Armonk, NY, USA). This retrospective study obtained local Institutional Review Board approval (401/2023 n. 186591).

## 3. Results

Between 1 February 2015 and 31 August 2022, 146 patients underwent open RC and met the inclusion criteria: 80 (54.8%) received conventional dressings (Group 1) and 66 (45.2%) received prophylactic iNPWT (Group 2). Most patients (97.0%) were treated for bladder cancer—predominantly muscle-invasive urothelial carcinoma—while a minority underwent RC for post-radiation cystopathy, bladder infiltration by prostatic adenocarcinoma, or cervical cancer. An ileal conduit urinary diversion, according to Bricker’s technique, was created in 76.7% of cases; a neobladder was fashioned in one case. Procedures were class II (clean-contaminated) in 96.0% of operations; five procedures were classified as contaminated due to rectal involvement or intraoperative spillage. Three patients required emergency RC for uncontrollable bleeding.

Preoperative and demographic data of patients are reported in Table 1. Baseline characteristics were broadly similar between the two groups. The mean age was 72.9 ± 8.4 years, and 70.5% were male. The mean BMI was 25.9 ± 3.9 kg/m^2^. Comorbidities were common among the study population, with a higher prevalence of CKD in the iNPWT cohort. ASA class was ≥3 in 57% of patients, and mean CCI was ≥5 in both groups. Notably, the iNPWT cohort had higher rates of CKD and prior laparotomic surgery and a greater proportion of active smokers, underscoring the need for adjusted analyses.

Risk factors for 90-day SSCs included higher BMI (mean 28.9 vs. 25.2 kg/m^2^, *p* = 0.001) and obesity (BMI > 30 kg/m^2^, *p* = 0.001), higher anesthetic risk (*p* = 0.03), prior open abdominal surgery (*p* = 0.01), and uncontrolled diabetes mellitus (*p* = 0.043). With respect to operative factors, longer procedures were associated with SSCs (mean 337 ± 20 vs. 310 ± 9 min, *p* = 0.02; operative time > 300 min, *p* = 0.04). No significant association was observed between SSCs and diversion type or estimated blood loss. Patients with SSCs experienced longer hospital stays (25.6 vs. 18.5 days, *p* = 0.03). From a pathological point of view, no differences were found in terms of the pathological stage of the disease or the presence of histological variables between the two groups. Detailed data are reported in Table 2.

Univariate analysis of intraoperative variables and risk of 90-day SSCs is detailed in Table 3. The primary outcome favored iNPWT: SSCs within 90 days occurred in 7.6% of the iNPWT cohort versus 22.5% in the conventional dressing group (*p* = 0.03). Surgical incision complications are reported in Table 4. Univariate logistic regression estimated a markedly lower odds of SSCs with iNPWT (odds ratio [OR] 0.282, 95%CI [0.099–0.808]), consistent with a protective effect. Of the 23 SSCs observed within 90 days, 21 (91.3%) were superficial and managed conservatively, whereas 2 (8.7%) represented deep fascial dehiscence requiring reoperation.

Viewed through a precision-medicine lens, the observed risk factors (obesity, diabetes, CKD, and prolonged operative time) provide actionable criteria for tailoring postoperative wound management. We calculated the Number Needed to Treat (NNT), which was equal to 6, demonstrating that even though the number of cases is not large, there may be clinical benefits even in small cohorts of patients, supporting selective application in patients with high-risk profiles.

Multivariate logistic regression confirmed that the use of iNPWT was independently associated with a reduced risk of surgical site complications at 90 days (Table 5). After adjustment for operative time, obesity (BMI ≥ 30), smoking status, previous abdominal surgery, acute myocardial infarction, cerebrovascular disease, COPD, CKD, and diabetes, iNPWT remained a significant protective factor (HR 0.22, 95% CI 0.06–0.78, *p* = 0.019). Diabetes also emerged as an independent predictor of complications (HR 3.44, 95% CI 1.17–10.14, *p* = 0.025). The overall model showed a significant improvement in fit compared with the null model (ΔΧ^2^ = 16.021, *p* = 0.099), with acceptable explanatory capacity (McFadden R^2^ = 0.099; Nagelkerke R^2^ = 0.126). These findings suggest that, beyond univariate associations, iNPWT exerts a measurable and independent effect in reducing wound morbidity, particularly in the presence of metabolic comorbidities such as diabetes.

## 4. Discussion

In our retrospective cohort, prophylactic iNPWT was associated with a clinically and statistically significant reduction in SSCs after open RC. This effect was observed alongside recognized risk factors—including obesity, diabetes, and prolonged operative time—that are frequently encountered in RC practice. Although most complications were found to be superficial and did not require reintervention, both from a psychological point of view and in terms of prolonging hospital stays, the non-use of the PICO^®^ system also had an impact on a small group of patients. Our findings align with accumulating evidence from abdominal and colorectal surgery demonstrating that closed-incision negative-pressure therapy reduces infectious and wound-related complications [7,8,9,12,13]. In the multivariate model, iNPWT remained an independent protective factor even after adjustment for major clinical variables, confirming its role beyond simple univariate associations. Conversely, diabetes emerged as an independent predictor of surgical site complications, underscoring the influence of metabolic factors on postoperative wound healing. These findings support the integration of iNPWT into selective, risk-based protocols rather than its uniform application to all patients.

Among the most representative works in the literature we find a systematic review by Ingargiola et al., published back in 2013, indicating that the use of this type of technology, although modified over time, remains a key point for patient and resource management [14]. Two other important meta-analyses are represented by the works of Hodgetts et al. and Scalise at al., who analyzed the available literature on the topic up to 2016 and both of whom concluded that the systematic use of iNPWT showed a decrease in the incidence of infection, sero-hematoma formation, and re-operation rates [12,13].

The contribution to personalized care is exploratory and hypothesis-generating: readily available risk factors (BMI, diabetes control, CKD, operative duration) may help target iNPWT selectively in high-risk patients, a premise that warrants prospective validation. Such a strategy is compatible with Enhanced Recovery After Surgery (ERAS) programs and supports individualized bundles rather than a one-size-fits-all approach [15,16]. When discussing minimally invasive procedures, it is essential to mention that robotic cystectomy is now playing an increasingly important role in the treatment of patients, leading to results that are similar from an oncological point of view but much better in terms of functional recovery. In this regard, several studies have compared surgical wound complications between open and robotic radical cystectomies, reporting significantly lower complication rates with the robotic approach [17,18]. Because wound morbidity differs between open and robotic approaches in several series, the present analysis intentionally focuses on open RC, where incisional complications remain clinically consequential and iNPWT is most directly applicable.

The biological plausibility of iNPWT is well established: by exerting uniform subatmospheric pressure, the device reduces lateral shear at the incision, evacuates exudate, limits edema, promotes perfusion, and may decrease bacterial bioburden. These mechanisms are pertinent in RC given extensive dissection, prolonged operative time, and the proximity of urinary stomas. The single-use, portable nature of PICO^®^ may facilitate adherence and outpatient continuation without impeding stoma care [19].

Implementation considerations include patient selection, device placement relative to stomas, and coordination with ostomy nursing to avoid effluent contamination. Economic aspects are relevant: while device costs are nontrivial, SSCs substantially extend length of stay and increase total treatment costs after RC, suggesting that targeted use of iNPWT could be cost-effective in high-risk subgroups [20,21,22,23]. In support of this, we report a recent systematic review of the literature on the iNPWT system published in 2024 by Formosa et al., which also confirms the usefulness of iNPWT on high-risk closed laparotomy incisions from a nursing perspective [24].

Strengths of this study include standardized perioperative care, a single-surgeon series minimizing technical variability, and clinically meaningful follow-up to 90 days. Limitations include the retrospective design, modest sample size, potential era-related confounding, incomplete capture of nutritional status, and the long period of recruitment, with the well-known technological changes that have taken place over time. A central limitation is a baseline imbalance between the groups (CKD, prior laparotomic surgery, smoking), introducing potential confounding by indication. Era effects are also possible because iNPWT was adopted from May 2019; although we adjusted for era in multivariable models, residual confounding cannot be excluded. Furthermore, the retrospective dataset did not consistently distinguish CDC-defined Surgical Site Infections from non-infectious dehiscence, necessitating a composite outcome for Table 4; we therefore report severity (superficial vs. deep) and management (reoperation vs. conservative care) as complementary descriptors. Collectively, these factors preclude causal inference and should be addressed in prospective, risk-stratified trials. The predominance of ileal conduit diversions may limit generalizability to continent reconstructions. Prospective randomized trials in RC, with prespecified risk stratification and health-economic analysis, are warranted to validate patient selection criteria and quantify cost-effectiveness.

## 5. Conclusions

Prophylactic closed-incision negative-pressure therapy using a single-use device (PICO^®^) reduced 90-day SSCs compared with conventional dressings after open radical cystectomy, and we should especially encourage its use in patients with elevated BMI, suboptimally controlled diabetes, CKD, or anticipated prolonged operative time. This tailored approach may improve outcomes, shorten hospitalization, and reduce costs. Future prospective studies should confirm these findings, refine risk thresholds, and evaluate quality-of-life and economic endpoints.

## Figures and Tables

**Table 1:** Preoperative and demographic characteristics of non-iNPWT vs. iNPWT patients.

Variable	Standard (*n* = 80)	iNPWT (*n* = 66)	*p*-Value
Age ± SD	72.3 ± 2.1	73.5 ± 2.2	0.65
Sex, *n* (%)			0.33
Male	58 (72.5)	45 (68.2)	
Female	22 (27.5)	21 (31.8)	
BMI (kg/m^2^)			
BMI	25.9	25.1	0.21
BMI ≥ 30, *n* (%)	16 (20.0)	6 (9.0)	0.20
Smokers, *n* (%)			
Active	22 (27.5)	33 (50.0)	0.013
Former	39 (48.8)	13 (19.7)	0.001
Charlson Comorbidity Index	5.9	6.6	0.48
ASA score			
ASA	2.6	2.6	0.51
≤2 pt, *n* (%)	35 (43.8)	28 (42.4)	
≥3 pt, *n* (%)	45 (56.2)	38 (57.6)	
Previous abdominal surgery, *n* (%)			
Laparotomic	27 (33.7)	37 (56.0)	0.019
Laparoscopic	2 (2.5)	1 (1.5)	0.015
Previous pelvic RT, *n* (%)	12 (15.0)	0 (0.0)	0.004
Neoadjuvant CT, *n* (%)	4 (5.0)	7 (10.6)	0.09
Acute myocardial infarction, *n* (%)	16 (20.0)	23 (34.8)	0.08
Cerebrovascular disease, *n* (%)	4 (5.0)	10 (15.2)	0.09
COPD, *n* (%)	10 (12.5)	13 (19.7)	0.31
CKD, *n* (%)	13 (16.3)	23 (34.8)	0.02
Diabetes, *n* (%)	18 (22.5)	17 (25.8)	0.67

BMI = body mass index; ASA = American Society of Anesthesiologists; RT = radiotherapy; CT = chemotherapy; COPD = chronic obstructive pulmonary disease; CKD = chronic kidney disease. Variables are reported as averages unless otherwise stated.

**Table 2:** Univariate analysis of clinical variables and risk of SSCs at 90 days after RC surgery.

Variable	No SSCs (*n* = 123)	SSCs (*n* = 23)	*p*-Value
Age	72.5	73.2	0.72
Sex, *n* (%)			0.12
Male	89 (72.4)	14 (60.9)	
Female	34 (27.6)	9 (39.1)	
BMI (kg/m^2^)			
BMI	25.2	28.9	0.001
BMI ≥ 30, *n* (%)	12 (9.8)	10 (43.5)	0.001
Smokers, *n* (%)			0.19
Active	43 (35.0)	9 (39.1)	0.81
Former	44 (35.7)	11 (47.8)	0.36
Charlson Comorbidity Index	6.1	6.4	0.51
ASA score	2.5	2.8	0.03
Anticoagulant therapy, *n* (%)	9 (7.3)	2 (8.7)	0.99
Antiplatelet therapy, *n* (%)	34 (27.6)	5 (21.7)	0.33
CKD, *n* (%)	31 (25.2)	5 (21.7)	0.38
Oral antidiabetic drugs, *n* (%)	15 (12.2)	10 (43.5)	0.001
Previous abdominal laparotomic surgery, *n* (%)	43 (35.0)	10 (43.5)	0.01
Previous pelvic RT, *n* (%)	8 (6.5)	4 (17.4)	0.23
Neoadjuvant CT, *n* (%)	9 (7.3)	2 (8.7)	0.99

SCCs = surgical site complications; BMI = body mass index; ASA = American Society of Anesthesiologists; CKD = chronic kidney disease; RT = radiotherapy; CT = chemotherapy. Variables are reported as means unless otherwise stated.

**Table 3:** Univariate analysis of intra- and perioperative variables and risk of SSCs at 90 days after RC surgery.

Variable	No SSCs (*n* = 123)	SSCs (*n* = 23)	*p*-Value
Surgical RC procedure, *n* (%)			
Bricker ileal conduit	91 (74.0)	21 (91.3)	0.09
Cutaneous ureterostomy	31 (25.2)	2 (8.7)	0.21
Neobladder	1 (0.8)	0 (0.0)	
Lymphadenectomy, *n* (%)			
Loco-regional	45 (36.6)	10 (43.5)	0.41
Extended	71 (57.7)	9 (39.1)	0.2
Surgical wound classification, *n* (%)			
Clean-contaminated	119 (96.7)	22 (95.7)	0.99
Contaminated	4 (3.3)	1 (4.3)	0.99
Emergency RC, *n* (%)	1 (0.8)	2 (8.7)	0.23
Operative time (min)			
Mean ± DS	310 ± 9	337 ± 20	0.02
≥300, *n* (%)	67 (54.5)	20 (87.0)	0.04
Estimated blood loss (mL)			
Mean ± DS	781 ± 95	1021 ± 376	0.21
≥500, *n*	75 (61.0)	21 (91.3)	0.15
Blood transfusion, *n* (%)	48 (39.0)	13 (56.5)	0.49
Subcutaneous synthesis, *n* (%)			
Not closed	2 (1.6)	0 (0.0)	0.18
Detached dots, single layer	116 (94.3)	21 (91.3)	0.54
Detached dots, double layer	1 (0.8)	2 (8.7)	0.08
Intradermal	4 (3.3)	0 (0.0)	0.99
Skin synthesis, *n* (%)			
Staples	123 (100)	22 (95.7)	0.18
Detached spots	0	1 (4.3)	
Drainage, *n* (%)			
Single	21 (17.0)	3 (13.0)	
Double	102 (83.0)	20 (87.0)	
Wound medication n (%)			
iNPWT	61 (49.6)	5 (21.7)	0.03
Conventional	62 (50.4)	18 (78.3)	0.17
LOS (days)	18.5	25.6	0.03

RC = radical cystectomy; iNPWT = incisional negative-pressure wound therapy; LOS = length of stay. Variables are reported as means unless otherwise stated.

**Table 4:** Surgical incision complications of non-iNPWT vs. iNPWT patients.

Incision Complication	Standard (*n* = 80)	Inpwt (*n* = 66)	*p*-Value
Hematoma, *n* (%)	0 (0.0)	0 (0.0)	/
Seroma, *n* (%)	0 (0.0)	1 (1.5)	0.78
Skin dehiscence/infection, *n* (%)	16 (20.0)	4 (6.0)	0.08
Fascial dehiscence, *n* (%)	2 (2.5)	0 (0.0)	0.50
Total *n* (%)	18 (22.5)	5 (7.6)	0.03

**Note:** In this retrospective dataset, skin dehiscence/infection was captured as a composite field; standardized CDC-defined SSI versus non-infectious dehiscence were not reliably distinguishable on chart review for all cases (see Limitations).

**Table 5:** Multivariate logistic regression for predictors of 90-day surgical site complications.

Variable	Hazard Ratio (95% CI)	*p*-Value
iNPWT	0.223 (0.063–0.784)	0.019
Operative time > 300 min	1.007 (0.983–1.031)	0.569
BMI ≥ 30	0.456 (0.108–1.935)	0.287
Smoker	1.410 (0.505–3.934)	0.512
Previous abdominal surgery	0.611 (0.208–1.795)	0.370
Acute myocardial infarction	0.734 (0.212–2.534)	0.624
Cerebrovascular disease	3.907 (0.881–17.324)	0.073
COPD	1.224 (0.325–4.604)	0.765
CKD	0.954 (0.289–3.142)	0.938
Diabetes	3.440 (1.168–10.135)	0.025

iNPWT = incisional negative-pressure wound therapy; BMI = body mass index; COPD = chronic obstructive pulmonary disease; CKD = chronic kidney disease; CI = confidence interval.

## Data Availability

The data that support the findings of this study are available on request from the corresponding author.

## Abbreviations

ASA	American Society of Anesthesiologists
BMI	Body Mass Index
CCI	Charlson Comorbidity Index
CDC	Centers for Disease Control and Prevention
CKD	Chronic Kidney Disease
CI	Confidence Interval
ERAS	Enhanced Recovery After Surgery
iNPWT	incisional Negative-Pressure Wound Therapy
OR	Odds Ratio
RC	Radical Cystectomy
SSC(s)	Surgical Site Complication(s)
pTNM	pathological Tumor–Node–Metastasis
